# Response to letters from Ms Deepa Bose (SAC) & Mr Alexander Thomas Schade (BOTA)

**DOI:** 10.1308/rcsann.2025.0109

**Published:** 2026-01-12

**Authors:** T Barrow, BD Chatterton, T Crompton, NT Kiely, SN Maripuri, K James

**Affiliations:** ^1^ Royal Alexandra Children's Hospital, UK; ^2^ Royal Sussex County Hospital, UK; ^3^ Robert Jones and Agnes Hunt Orthopaedic and District Hospital NHS Trust, UK

Dear Editor,

We would like to thank the authors, on behalf of the Specialist Advisory Committee (SAC) in Trauma and Orthopaedics and the British Orthopaedic Trainee Association (BOTA), for their considered responses to our paper on operative experience in paediatric orthopaedics in UK trainees.^1^

We are grateful for the clarification from the SAC regarding the distinction between curriculum requirements at Level 4 and procedure-based assessments. We agree that competence in managing a condition does not necessarily equate to independently performing all surgical procedures associated with its management.

However, our data do suggest there is limited experience in managing some critical conditions related to paediatric orthopaedic care that may constitute true surgical emergencies. An example highlighted is musculoskeletal infection, the surgical management of which might be expected by a general orthopaedic consultant in a non-tertiary centre, a point also made by the BOTA group.

The BOTA response states the paper does not report how often trainees assist in key procedures. These data are included in the paper, and we would draw the authors’ attention to the supplementary material of the article, available online (this table is called out in the text as Table S1). These data demonstrate a median case volume of 0 for assisting in slipped upper femoral epiphysis pinning or open reduction and internal fixation, and arthroscopic or open washout of infected joints (shoulder/hip/knee/ankle).^1^ We agree there are differing management options, such as arthrocentesis or arthroscopy, for musculoskeletal infection in children; however, the data suggest that trainees also have limited experience in these alternatives. Overall, we acknowledge that the urgent surgical management of these conditions is rare and therefore challenging for trainees to experience during training. We note in our paper that different training modalities such as simulation may be required for these rare emergencies, as noted by BOTA.

We acknowledge the dataset lacks granular data with relation to regional geography or treating hospital. Unfortunately, this is a stipulation of the Joint Committee on Surgical Training (JCST) in providing eLogbook data for understandable reasons of confidentiality. We agree that workplace-based assessments represent a valuable component of surgical training, although these were outside the scope of this study and not included in the dataset. We are fully in agreement that simply counting operative numbers is not an indicator of competence, and curricula must evolve to reflect this.

We agree wholeheartedly on the importance of regional networks and specialist centres in delivering care to paediatric patients, particularly for rarely encountered conditions in training such as slipped upper femoral epiphysis, which may have significant long-term sequalae. We believe it is vitally important that it is clearly defined in networks which conditions require management in specialist tertiary centres and which can be managed locally. There is large national variance in the management of paediatric orthopaedic conditions, particularly in trauma practice, and stronger regional protocols and equitable exposure to specialist paediatric orthopaedic training could aid in reducing such variability.^2^

The authors would like to thank the SAC and BOTA for engaging with our work and for their continued commitment to high-quality orthopaedic training. We hope that the findings of our study contribute to the discussion about how best to support trainees in developing the skills required for safe and independent practice.

## Competing interests

The author/s declare no competing interests.

## Funding

The author/s received no financial support for the research, authorship and/or publication of this article.

## Author contributions

**T Barrow:** Conceptualization, Data curation, Formal analysis, Writing – original draft, Writing – review & editing. **BD Chatterton:** Conceptualization, Data curation, Formal analysis, Writing – original draft, Writing – review & editing. **T Crompton:** Writing – review & editing. **NT Kiely:** Writing – review & editing. **SN Maripuri:** Writing – review & editing. **K James:** Conceptualization, Writing – original draft, Writing – review & editing.

## Artificial Intelligence

The author/s declare that no AI was used to conduct the study or prepare the manuscript.
